# Effects of implementing the act of prohibition on sex trafficking on female sex workers’ sexually transmitted infections

**DOI:** 10.1371/journal.pone.0182465

**Published:** 2017-08-09

**Authors:** Minsoo Jung

**Affiliations:** Department of Health Science, Dongduk Women's University, Seoul, South Korea; University of Illinois at Chicago, UNITED STATES

## Abstract

This study investigated the effect of implementing the act of prohibition on sex trafficking (PST) on sexually transmitted disease (STD) infections among South Korean female sex workers (FSWs) working at prostitution blocks. Research data were collected twice through the Korean government-sanctioned survey for female sex workers (1^st^ wave = 1,083; 2^nd^ wave = 926). We examined the associations among health behavior, working conditions, and the effect of PST act via hierarchical logistic regression analyses using propensity score matching. After adjusted covariates, the risk probability was 0.288 times lower among FSWs who had remained in prostitute blocks after the PST act enforcement compared to FSWs who had worked before the PST. Similarly, the risk probability for a gonorrhea infection was 0.219 times lower among FSWs who had remained in prostitute blocks after the PST act compared to FSWs who had worked before the PST. Therefore, this study showed that, besides already known factors, the implementation and establishment of the PST Act was a strong factor that suppressed STD infections among FSWs.

## Introduction

In 2005, South Korea implemented the Act of Prohibition on Sex Trafficking (PST Act). Before the legislation of this law, the nation had 270 thousand female sex workers (FSWs) working at approximately 45,400 brothels with prostitution industry amounting to 14 trillion won, i.e., 12.68 billion USD [1]. Prostitution in established locations is largely divided into two types. Establishments with full-time female sex workers (FSWs) mainly deal with sex trafficking. They are densely populated in red-light districts (RLDs). In these areas, FSWs maintain their livelihood exclusively through prostitution (3,600 FSWs working at approximately 1,400 brothels). In contrast, establishments that offer prostitution as a side-business mainly sell alcohol or massage services. They try to supplement their business by also offering prostitution (270,000 FSWs working at approximately 44,000 brothels). In the end, the country’s illegal sex industry was immense. Approximately 1.07% of its total female population working in the sex industry, with prostitution reaching 1.7% of the gross domestic product (GDP) of South Korea [2].

In the nation’s industrialization process after liberation from Japanese colonial rule (1910–45), the sex industry lived off the South Korean society and grew as part of urban centers. Establishments with full-time FSWs exhibit typical and consistent sexual behaviors that differ from those of FSWs in situations where they directly or indirectly make contact with clients and form relationships not at a set location. Brothels are generally located at the periphery of busy downtown areas with a high volume of population movement. RLDs usually have about 10 to 50 brothels located in close proximity to each other. They solicit clients as a group. Each brothel usually houses about 2 to 10 FSWs who live with their pimps and stay there for a long time. However, with incidents such as the one where FSWs managed under confinement by brothel owners all lost their lives in a fire, adverse human rights circumstances and exploitation of these women became controversial starting from 2000. The PST Act was legislated as a result. The numbers of brothels and FSWs have been decreased by 1/3 and 1/5, respectively, over one decade since then (Lee & Lee 2010). However, the actual effectiveness of the PST Act on sexually transmitted diseases (STDs) among FSWs has not been reported.

In fact, the PST Act has given rise to countless ongoing controversies and arguments in diverse quarters of society not only in its legislation process, but also in its implementation process [3–6]. One of the main reasons is that, since the implementation of this law, violations have not been properly controlled in many cases. Consequently, since 2009, the South Korean government has taken administrative measures by demolishing prostitution blocks and subjecting these areas to urban redevelopment [6]. In response, brothel owners and FSWs have staged mass demonstrations, citing their right to life, and vehemently resisted against demolition. At the same time, some FSWs, feeling threatened, have left prostitution blocks and chosen part-time prostitution which is even more difficult to control. This includes selling sex secondarily at karaoke bars and massage parlors or engaging in prostitution at venues such as “officetels” through social networking services (SNS). In other words, in South Korea, part-time FSWs commute to entertainment establishments from their own homes, serving alcohol at the table, and engage in sex trafficking at work. In contrast, full-time FSWs lodge at brothels, living together with other FSWs under the management of the brothel keepers for periods ranging from several months to several years.

Such resistance to the PST Act has given rise to two problems in terms of STD control. First, there has been the so-called balloon effect, i.e., while full-time FSWs at prostitution blocks have decreased, part-time FSWs have increased. Consequently, it is difficult to state conclusively that prostitution itself has been suppressed through the PST Act. Second, prostitution blocks have become illegal spaces in the confrontation between police and FSWs during their demolition, leading to interruption to medical checkups for STDs hitherto implemented on FSWs by the state. As a result, it has become even more difficult to calculate STD prevalence rates among FSWs. We investigated the effect of the implementation of the PST Act on STD infections among South Korean FSWs working at prostitution blocks. In particular, we examined the factors affecting STD infections among FSWs who had been working at prostitution blocks in 2008 before their demolition and those who continued to remain at prostitution blocks in 2014 even after the demolition by over that has been done for more than 50%. We explored whether the PST Act had contributed to the prevention of STD infections among full-time FSWs.

## Methods

### Study design

We conducted a comparative national survey on 16 representative sex work venues (i.e., red light districts) twice (2008 and 2014) through collaboration with the Korea Federation for HIV/AIDS Prevention (KAIDS). Though the PST Act took effect in 2005, it was not until nearly 2010 that the number of sex-trafficking establishments in RLDs actually and gradually declined. Therefore, we conducted a comparative study at two points in time: 2008 and 2014. Out of the datasets from the two time points used in this study, those for 2008 were published in a range of papers. However, the 2014 datasets have not yet been published in any paper. Likewise, the 2008 datasets used in this study differ from those in earlier papers in terms of the statistical analysis methods and the research model, which makes this study amply unique.

### Sample

FSWs were recruited using a multistage stratified cluster random sampling method applied to time location sampling (TLS). TLS is appropriate for representative sex workers who are easily located at visible and designated sites. It has been used to sample sex workers who are accessible at public locations listed in a sampling frame [7, 8]. For this study, the 42 national RLDs listed by the Ministry of Gender Equality and Family were stratified according to city population and brothel size. A total of 16 areas were selected. Sample size determination was based on the assumption that the target FSW population was 4,500 which was reported in 2008 by the Korean Ministry of Gender Equality. Based on that estimate, the required sample size was 800 FSWs, assuming chlamydia prevalence of 30% with a precision of 2.5% [9, 10].

### Data collection

Research data were collected twice through the Korean government-sanctioned survey for female sex workers. The first survey was conducted in 2008 with 1083 respondents (1^st^ wave). The second survey was conducted in 2014 survey with 926 respondents (2^nd^ wave). We recruited representative brothel owners of 24 sex work venues. They were identified in a nationwide economic survey of the sex industry in 2008 [9]. Sample size was calculated based on the estimation from the Gender Policy Research Institute report of 2008. Individual surveys were conducted through 15 minute interviews administered by a team consisting of a physician, two clinical pathologists, an individual with a master degree in public health, and a field director. Laboratory technicians obtained urine and blood samples from FSWs. Trained interviewers encouraged FSWs to answer the questionnaire on health status. Biological samples were obtained for the following tests: serum TP-PA test for *T*. *pallidum* and urine PCR test for *N*. *gonorrhoeae* presence. The total response rate for the two surveys was 92.0% for the 2008 survey and 93.4% for the 2014 survey. After manual reviewing the completed questionnaires, data were input via a double entry system. Urine samples were analyzed at a central laboratory with a commercial PCR kit (BDProbTecET, BD Diagnostic Systems). A pathologic test for clinical specimens was conducted at a community health center near each survey district and the Seoul Medical Science Institute, South Korea.

### Measures

The measurement used for each study variable was based on those described in the abridged version of the Behavioral Surveillance Surveys, an internationally validated survey approach [11].

#### Dependent variables

With regard to STD infection, all participants currently infected with active syphilis and gonorrhoeae were included. Current infection was identified through clinical examinations. Past infection was identified by physicians through survey questioning conducted during participant examination. STD infection question responses were coded as ‘1’ if pertinent results were positive or ‘0’ if pertinent results were negative.

#### Independent variables

Health-related characteristics: For smoking, we asked the following question: “How many cigarettes do you smoke a day?” We measured this item with a 5-point scale based on the frequency of smoking. Response options were collapsed into the following categories: two packs or more, one pack, half pack, half pack or less, or does not smoke. Physical health of FSW was measured by self-rated health (SRH) status. We asked the following questions: “In general, would you say your health is very good, good, average, poor, or very poor? Although such rating simplifies a complex issue, it has been used to reliably predict survival in populations even after taking into account other known health risk factors [12]. It has also been validated as a good predictor for morbidity and mortality [13]. Depression was assessed through asking the following question: “Have you ever experienced depression in the past month?” The response options were yes or no. To reduce social desirability bias, we used validity checks to measure internal consistency.

Sexual risk characteristics: Age at sexual initiation was determined by asking whether participants had their first sexual intercourse experience. We also measured sexual risk behaviors with the number of customers and the frequency of condom use with customers. The number of customers was determined by asking a closed-ended question, “How many customers did you have per day on average?” The frequency of condom use was assessed by asking the following question: “How often did you use condoms with your customers?” The answer was collapsed into the following categories: ‘over 90%’, ‘70%-90%’, ‘50%-70%’, or ‘under 50%.’ For analysis, the original scale was categorized. In addition, those who were previously infected and diagnosed with STD were included.

PST enforcement: The circumstances in 2008 before the manifestation of the effect of the PST Act were compared to those in 2014 when a considerable number of major prostitution blocks nationwide had been shut down and the control of illegal prostitution had taken roots through the implementation of the PST Act. For analysis, we recorded the year of 2008 as (0) and the year of 2014 as (1). They were entered into the regression model as a dummy variable.

#### Covariates

Sociodemographic covariates were selected based on their theoretically and empirically defined relationships with social determinants of STD infections [9, 14]. Age was divided into the following categories: < 19, 20–25, 26–30, 31–35, 36–40, and 41 years or older. Educational attainment was assessed using the following question: “What is the highest level of school that you have attended?” The response was categorized into ‘elementary school’, ‘middle school’, ‘high school’, and ‘college or higher.’ We also adjusted the recruitment region for the sample.

### Statistical analyses

We created matched sets of treated and untreated participants using a predicted probability of a propensity score in order to minimize total within-pair difference [15]. We estimated the treatment effect, i.e., before and after the PST act, by comparing dependent variables between the treated group and the untreated group in the matched sample (n = 830). Statistical analyses procedure was as follows. First, we estimated the descriptive statistics of the survey participants’ prevalence of active syphilis and *Neisseria gonorrhoeae* from the two national samples. Independent sample t-tests were used to examine the associations among individual factors and the time difference between 2008 and 2014. This method can be used when the effect of the independent variable on the dependent variable is influenced by covariates. Second, Mantel-Haenszel X^2^ test allowing the comparison of two groups for a dichotomous/categorical response was used to compare the associations between STD infection and the time difference before and after the PST act. Third, we examined the associations among health behavior, working conditions, and the effect of the PST act via hierarchical logistic regression analyses. We sequentially entered all study variables identified as significant factors by previous studies into the model [9]. To adjust for potential biases in recruitment, we weighted the data by inverse of the approximate probability of recruitment [16]. All statistical analyses were performed using STATA version 12 (STATA Corp, College Station, TX). Participant responses with missing values for key analysis variables were excluded using a pairwise method.

### Ethics statement

The initial research (1^st^ wave) passed the institutional review board of Seoul National University Hospital in May 14th, 2008 (No: C-0801-047-232). Regarding 2^nd^ wave survey, ethics and governance approvals were awarded by the Institutional Review Board, Korea Centers for Disease Control and Prevention (January 22, 2014; 20140122). In order to protect vulnerable research subjects, actual site interviews and investigations were performed after receiving written informed consent from the participants. During the investigation process, absolutely no information that could identify individual respondents was collected.

## Results

### Comparison of social characteristics among two samples before and after the PST act

FSWs working at prostitution blocks in 2008 (n = 1,083) and 2014 (n = 926) were recruited. Their general characteristics are shown in Table 1. The ages of these women were concentrated in the range of 26 to 30 years (44.4%) in 2008. However, they were concentrated in the range of 20 to 25 years (32.9%) in 2014, with legal minors under the age of 19 being 11.0%. The decrease in average age was approximately two years, which was statistically significant (*p* < 0.001). However, there was no statistically significant change in the age of first sexual experience. Regarding educational attainments, at both time points (2008 and 2014), high school graduates accounted for approximately 73%, constituting the majority among FSWs. Compared to those FSWs in 2008, the number of highly educated women with college/university-level diplomas or above was increased by nearly twice (from 8.9% to 15.5%, *p* < 0.001) in 2014. Meanwhile, health behavior worsened as well. The percentage of non-smokers was decreased from 23.3% in 2008 to 12.1% in 2014. The percentage of smokers who smoked one or more packs of tobacco per day was dramatically increased from 10.8% in 2008 to 72.8% (*p* < 0.001) in 2014. However, self-rated health status reported by these respondents was improved slightly but significantly (*p* < 0.001). Regarding the prostitution environment, the average number of sex clients per day was increased (*p* < 0.001) by approximately 0.9 in 2014 compared to that in 2008. However, there was no statistically significant change in condom use rate between the two time points.

**Table 1:** Comparison of social characteristics among two samples before and after the PST act.

		2008 (n = 1083)		2014 (n = 926)		t (p-value)
		Frequency	%	Frequency	%	
Age	19 or under	5	0.5	100	11.0	6.200
	20–25	306	28.5	300	32.9	(p<0.001)
	26–30	476	44.4	316	34.7	
	31–35	177	16.5	111	12.2	
	36–40	58	5.4	43	4.7	
	41 or older	50	4.7	41	4.5	
Education	Elementary school or less	18	1.7	12	1.5	-5.168
	Middle school	173	16.2	81	9.9	(p<0.001)
	High school to associate	782	73.2	600	73.2	
	College or higher	95	8.9	127	15.5	
Smoking (per day)	Don’t smoke	251	23.3	105	12.1	-28.166
	Half pack or less	581	54.0	37	4.3	(p<0.001)
	Half pack	128	11.9	93	10.8	
	One pack	31	2.9	437	50.5	
	Two packs or more	85	7.9	193	22.3	
Self-rated health status (SRH)	Very poor	11	1.0	5	.6	-3.210
	Poor	113	10.5	81	9.4	(p<0.001)
	Average	507	46.9	372	43.1	
	Good	365	33.8	297	34.4	
	Very good	84	7.8	109	12.6	
Depression	Yes	771	71.5	611	70.4	-0.546
	No	307	28.5	257	29.6	(n.s.)
Age at first sex (years)	15 or under	51	5.0	34	4.1	-1.077
	16–19	483	46.9	302	36.6	(n.s.)
	20–25	479	46.5	471	57.1	
	26 or older	17	1.7	18	2.2	
Number of customers (per day)	3 or less	517	57.2	283	41.1	-6.787
	4–6	267	29.5	301	43.7	(p<0.001)
	7–9	68	7.5	58	8.4	
	10 or more	52	5.8	47	6.8	
Frequency of condom use during last month	50% or less	564	53.4	473	55.2	1.495
	50%	155	14.7	141	16.5	(n.s.)
	70%	118	11.2	87	10.2	
	80%	150	14.2	111	13.0	
	90%	59	5.6	37	4.3	
	100%	10	.9	8	.9	

PST act: Act on Prohibition on Sex Trafficking; n.s., Not significant.

### Bivariate analyses of STD infections among two samples before and after the PST act

Results of bivariate analyses of STD infections are presented in Table 2. Among the 2008 samples, 9.7% were syphilis-positive [95% confidence interval (CI): 8.0%–11.6%] and 2.5% were gonorrhoeae-positive (95% CI: 1.6%–3.6%). Among the 2014 samples, 2.6% were syphilis-positive (95% CI: 1.5%–3.7%) and 1.0% were gonorrhoeae-positive (95% CI: 0.3%–1.7%). Significant differences in STD infection experience (*p* < 0.05) and types of STD (syphilis, *p* < 0.001; gonorrhea, *p* < 0.01) were found between the samples before the PST act and the samples after the PST act.

**Table 2:** Bivariate analyses of STD infections among two samples before and after the PST act.

		2008	2014	Total	X^2^ (P-value)
		Frequency (%)	Frequency (%)	Frequency (%)	
Experience of STD	Yes	750 (73.2%)	582 (68.6%)	1332 (71.1%)	4.654 (p<0.05)
	No	275 (26.8%)	266 (31.4%)	541 (28.9%)	
Syphilis	Negative	963 (90.3%)	831 (97.6%)	1794 (93.6%)	42.137 (p<0.001)
	Positive	103 (9.7%)	20 (2.4%)	123 (6.4%)	
	Prevalence rates	9.7% (95% CI: 8.0%-11.6%)	2.6% (95% CI: 1.5%-3.7%)		
N. gonorrhoeae	Negative	1013 (97.4%)	843 (99.1%)	1856 (98.1%)	7.066 (p<0.01)
	Positive	27 (2.6%)	8 (0.9%)	35 (1.9%)	
	Prevalence rates	2.5% (95% CI: 1.6%-3.6%)	1.0% (95% CI: 0.3%-1.7%)		

All prevalence rates were weighted by sample recruitments

STD: sexually transmitted disease; PST act: Act on Prohibition on Sex Trafficking

### Multivariable analyses of determinants of STD infections by enforcement of the PST act

The relationships between PST act enforcement and STD infections after adjusted for sociodemographic baseline factors including age, education, and recruitment region are shown in Tables 3 and 4. As shown in the Table 3, the probability of a syphilis infection was 2.040 times higher for FSWs who had STD infection experience (Model III; 95% CI: 1.312–3.173). However, the probability of a syphilis infection was 0.288 times lower among FSWs who had remained in prostitute blocks after the PST act enforcement compared to FSWs who had worked before the PAT (95% CI: 0.160–0.519). Similarly, the risk probability for an gonorrhea infection was 0.219 times lower among FSWs who had remained in prostitute blocks after the PST act compared to FSWs who had worked before the PAT (95% CI: 0.066–0.723). These results showed that the probability of STD infection was significantly associated with the effect of legal banning on prostitution and sex trafficking. However, there was no association among the likelihood of STD infection, working conditions, and health status of FSWs.

**Table 3:** Multivariable analyses of determinants of syphilis infections by enforcement of the PST act (n = 830).

	Model I				Model II				Model III			
	aOR	p-value	95% CI		aOR	p-value	95% CI		aOR	p-value	95% CI	
			Lower	Upper			Lower	Upper			Lower	Upper
Smoking	.860	.048	.741	.999	.871	.093	.742	1.023	1.035	.708	.865	1.239
SRH	1.113	.386	.874	1.416	1.199	.191	.914	1.573	1.228	.144	.933	1.616
Depression	.839	.450	.532	1.324	.828	.451	.508	1.351	.871	.583	.531	1.427
Age at first sex					.939	.219	.850	1.038	.950	.318	.859	1.051
STD experience					1.998	.002	1.288	3.099	2.040	.002	1.312	3.173
Number of customers					.978	.613	.898	1.065	.997	.948	.917	1.084
Condom Use					.996	.960	.851	1.165	.984	.844	.840	1.153
Year (Ref.: 2008)									.288	.000	.160	.519
Constant	34.990	.001			125.689	.011			245.107	.006		
-2 Log Likelihood	808.632				700.876				680.792			
Nagelkerke R^2^	.068				.086				.118			

PST act: Act on Prohibition on Sex Trafficking; aOR: adjusted odds ratio; SRH: self-rated health status; STD: sexually transmitted disease

All models are additionally adjusted for age and region after matching with propensity score.

**Table 4:** Multivariable analyses of determinants of N. gonorrhoeae infections by enforcement of the PST act (n = 830).

	Model I				Model II				Model III			
	aOR	p-value	95% CI		aOR	p-value	95% CI		aOR	p-value	95% CI	
			Lower	Upper			Lower	Upper			Lower	Upper
Smoking	.810	.119	.622	1.055	.732	.050	.537	.999	.918	.639	.642	1.313
SRH	1.097	.668	.720	1.671	1.070	.780	.665	1.722	1.106	.681	.684	1.789
Depression	1.965	.064	.961	4.016	1.832	.142	.817	4.105	1.903	.117	.851	4.257
Age at first sex					1.041	.661	.871	1.243	1.066	.487	.890	1.276
STD experience					.739	.505	.304	1.796	.775	.574	.319	1.883
Number of customers					.927	.328	.797	1.079	.951	.506	.821	1.102
Condom Use					1.198	.178	.921	1.559	1.206	.162	.928	1.567
Year (Ref.: 2008)									.219	.013	.066	.723
Constant	.004	.032			.001	.076			.000	.070		
-2 Log Likelihood	337.395				274.391				266.645			
Nagelkerke R^2^	.022				.050				.079			

PST act: Act on Prohibition on Sex Trafficking; aOR: adjusted odds ratio; SRH: self-rated health status; STD: sexually transmitted disease

All models are additionally adjusted for age and region after matching with propensity score.

## Discussion

STDs are contagious diseases. Their infection and transmission occur mainly through sexual contact. Policies and regulations on prostitution, which presuppose sexual intercourse, obviously can affect the spread of STDs. Because of the nature of their work, FSWs constitute a major mediating channel for STDs. They are vulnerable to STDs [17, 18]. Consequently, South Korea’s legislation of the PST Act on prostitution [19] is expected to have a considerable effect on STD infections among FSWs.

This study revealed that, through the implementation of the PST Act, STD infections among full-time FSWs in South Korea have changed over the past decade. First, STD prevalence rate among FSWs was decreased significantly in 2014 in comparison with that in 2008 before the manifestation of the effect of the PST Act. Prevalence rate of syphilis was decreased by 73% (from 9.7% in 2008 to 2.6% in 2014). The prevalence of gonorrhea was decreased by 60% (from 2.5% in 2008 to 1.0% in 2014). Second, after adjusting for covariates, the strongest factor that affected STD infections among FSWs was the introduction of the PST Act. Decreases in STD prevalence rates were affected not only by laws and institutions, but also by other personal and relational factors. However, the results of logistic regression in this study showed that, besides already known factors, the implementation and establishment of the PST Act was also a strong factor that suppressed STD infections among FSWs. After 2008, when a preliminary study was conducted nationwide, the PST Act raised the level of control and suppressed prostitutions throughout the society by phased demolition of prostitution blocks. Although some assessment have revealed that the PST Act’s actual effectiveness is low, this law has served as a possible preventive factor on STD infections among FSWs.

However, new problems were discovered through this study as well. First, in comparison with the 2008 survey, the 2014 survey showed a new influx of young and highly educated women into prostitution blocks despite a considerable reduction in the number of FSWs. FSWs generally leave prostitution blocks as they get older [6]. The results of the 2014 survey showed that the average age of the respondents was nearly two years younger compared to that of respondents in the 2008 survey, indicating that there was a new influx of women into the prostitution industry. Furthermore, compared to respondents in 2008, the respondents in the 2014 survey smoked far more and received an average of 0.9 more sex clients per day. Consequently, while FSWs’ STD infection rates were decreased, their health behavior and working environments seemed to have deteriorated further. This appeared to be a dilemma arising from the situation when prostitution was not eradicated completely. Consequently, change throughout the social structure is necessary to resolve the problem of STDs among FSWs at prostitution blocks fundamentally. For example, this can be achieved by controlling the supply and demand chain of prostitution by intensively managing prostitution procurers and male sex clients.

This study has several limitations. First, the gatekeeper characteristics of different RLDs and sex-traffic establishments surveyed in our study were not assessed [20]. Gatekeepers are usually brothel owners. They can implement different tacit norms for condom use and the number of daily clients. Variation in these norms might have affected our results. Second, we did not cover all types of FSWs. Our sample was limited to full-time FSWs. Differences between full-time and part-time FSWs may produce different results [21].

In the course of visiting prostitution blocks to study STDs among FSWs, one encounters the most vulnerable women in the society who can only make their livelihoods by selling sex. In nearly all cases, these women have been driven into prostitution by unemployment and destitution. The present study showed that the implementation of the PST Act contributed to the protection of these women’s physical health, although its contribution might be small. However, the problem is that prostitution is still practiced at prostitution blocks even though ten years have passed since the implementation of a law categorically prohibiting it. Moreover, it is very difficult to estimate STD infection rates in the case of secret prostitution. Nevertheless, there is no possibility that South Korean society can revert back to the days before PST Act. Consequently, it is necessary to determine the benefits and limitations of the PST Act to control STDs among FSWs.

## Abbreviations

CIs: confidence intervals
FSWs: female sex workers
KAIDS: Korea Federation for HIV/AIDS Prevention
ORs: odds ratios
RLDs: red-light districts
SRH: self-rated health
STIs: sexually transmitted infections
